# Changes in ultra-processed food consumption during the first Italian lockdown following the COVID-19 pandemic and major correlates: results from two population-based cohorts

**DOI:** 10.1017/S1368980021000999

**Published:** 2021-03-05

**Authors:** Marialaura Bonaccio, Emilia Ruggiero, Mariarosaria Persichillo, Simona Esposito, Marco Olivieri, Augusto Di Castelnuovo, Chiara Cerletti, Maria Benedetta Donati, Giovanni de Gaetano, Licia Iacoviello

**Affiliations:** 1 Department of Epidemiology and Prevention, IRCCS Neuromed, Via dell’Elettronica, Pozzilli (IS) 86077, Italy; 2 Mediterranea Cardiocentro, Napoli, Italy; 3 Associazione Cuore-Sano ONLUS, Campobasso, Italy; 4 Department of Medicine and Surgery, Research Center in Epidemiology and Preventive Medicine (EPIMED), University of Insubria, Varese, Italy

**Keywords:** COVID-19, Lockdown, Dietary changes, Ultra-processed food, Diet quality

## Abstract

**Objective:**

To evaluate changes in ultra-processed food (UPF) intake and its major correlates during the first Italian lockdown (9 March–3 May 2020).

**Design:**

Retrospective observational study.

**Setting:**

Italy.

**Participants:**

We analysed 2992 subjects (mean age 57·9 ± 15·3 years, 40·4 % men). Individual participant data were pooled from two retrospective cohorts: (1) The Moli-LOCK cohort consists of 1501 adults, a portion of the larger Moli-sani study (*n* 24 325; 2005–2010) who were administered a phone-based questionnaire to assess lifestyles and psychological factors during confinement and (2) the Analysis of Long Term Risk of Covid-19 Emergency is a web-based survey of 1491 individuals distributed throughout Italy who self-responded to the same questionnaire by using Google forms.

UPF was defined according to NOVA classification based on degree of food processing. An UPF score was created by assigning 1 point to increased consumption, −1 to decreased and 0 point for unchanged intakes of nineteen food items, with higher values indicating an increase in UPF during confinement.

**Results:**

Overall, 37·5 % of the population reported some increase in UPF (UPF score ≥1). Adults were more likely to decrease UPF (multivariable regression coefficient *β* = −1·94; 95 % CI −2·72, −1·17 for individuals aged >75 years as compared with 18–39 years) as did individuals from southern Italian regions as compared with Northern inhabitants (*β* = −1·32; 95 % CI −1·80, −0·84), while UPF lowering associated with increased exercise (*β* = −0·90; 95 % CI −1·46, −0·35) and weight loss (*β* = −1·05; 95 % CI −1·51, −0·59) during confinement.

**Conclusions:**

During the first Italian lockdown, about 40 % of our population switched to unfavourable eating as reflected by increased UPF intake and this may have long-term effects for health.

COVID-19 is caused by severe acute respiratory syndrome coronavirus 2 and was recognised by the WHO as a pandemic early in 2020^(1)^. As of 28 January 2021, there have been over 100 million of confirmed cases of COVID-19, with 2·177·677 deaths worldwide^(2)^.

The containment measures released by the Italian Government for the period between 9 March and 3 May 2020 (#stay at home decree) were aimed to reduce the movement of people from their homes, which was only allowed in cases of special need and for buying food and other basic supplies in nearby food stores and supermarkets; mass gatherings and events were completely banned, while the use of smart/home working was largely encouraged wherever possible^(3)^.

As a consequence, the lockdown period is likely to have lasting effects on people and their lifestyles, especially nutrition, and psychosocial well-being.

Previous evidence has shown that the main consequence of quarantine is a change in lifestyle and nutritional habits due to either a limited access to food caused by restricted store opening hours or as a result of psychological stress and boredom during confinement^(4)^. Psychological stress and boredom have been reportedly associated with overeating and consumption of comfort food and food craving^(5)^ which in turn may have adverse long-term health effects especially for cardiovascular health.

Accumulating evidence from studies conducted worldwide during the first wave of the COVID-19 pandemic indicated an increase in the intake of unhealthy foods (e.g. snacks, sugary drinks and chocolate), although a global assessment of changes in diet quality is often lacking with many studies reporting either increases or decreases of unhealthy foods^(6–8)^.

In last years, concerns have been raised on the potential health impact of ultra-processed foods (UPF) in the diet, whose consumption has increased dramatically over the last decades leading to a gradual substitution of traditional diets. UPF are made by industry processing or chemical synthesis from processed substances extracted or refined from whole foods; they are usually rich in additives used to imitate or enhance the sensory features of foods, such as colour stabilisers, flavour enhancers and non-sugar sweeteners^(9–11)^. Several population studies have provided evidence of a strong association between increased UPF intake and risk of major chronic diseases^(12,13)^ and mortality^(14,15)^, often independently of overall diet quality. Moreover, the dietary imbalance caused by regular consumption of UPF increases the inflammatory potential of the diet: this would negatively influence lung function^(16)^ and immunity^(17)^, possibly leading to an increased susceptibility to severe acute respiratory syndrome coronavirus 2^(17)^.

In view of this, it is crucial to understand whether and to what extent home confinement during the COVID-19 pandemic had an impact on consumption of UPF and which are the potential consequences on long-term health effects at population level.

The aim of the current study was to investigate the impact of the first Italian lockdown period on UPF consumption by analysing both changes in single food items and global modifications in diet quality and their major correlates by using data on 2992 men and women from two population-based cohorts recruited from May 2020 to September 2020.

## Methods

### Study design and participants

#### The Moli-LOCK study

The Moli-LOCK study was designed as an observational cohort study that retrospectively investigated dietary, lifestyle and psychosocial changes possibly occurred after Italy’s lockdown following the COVID-19 pandemic, that is, in the period of time between 9 March 2020 and 3 May 2020. The population of the Moli-LOCK study consists of a sub-group of men and women who had first been recruited in the larger Moli-sani study cohort^(18)^ in 2005–2010 (*n* 24 325) and then re-examined in 2017–2020 (*n* 2572). From May 2020 to September 2020, subjects were contacted by phone by trained researchers to be administered a questionnaire to assess lifestyle, dietary and psychosocial changes during the confinement following the COVID-19 pandemic. A total of 1563 completed the questionnaire, as compared with the eligible sample who did not participate (*n* 1009), individuals included in the study were slightly younger (66·4 ± 8·6 *v*. 67·5 ± 9·3; *P* value <0·001 for analysed *v*. excluded) and had higher education (upper secondary school or higher = 66·2 % *v*. 61·5 %, respectively, *P* = 0·015), while no differences were found for sex (men = 43·7 % *v*. 47·1 %, respectively, *P* = 0·09) and presence of chronic diseases (CVD = 7·4 % *v*. 9·3 %, respectively, *P* = 0·08; cancer = 9·4 % *v*. 9·2 %, *P* = 0·72). After exclusion of those participants with missing information on UPF intake (*n* 62), we finally analysed 1501 subjects. Online Supplementary Figure S1 shows the flowchart for selection of study participants.

#### The ALT RISCOVID-19 survey

The ALT RISCOVID-19 (Analysis of Long Term Risk of Covid-19 Emergency) was a cross-sectional web-based survey carried out among Italian adults aged ≥ 18 years, residing in Italy during the confinement.

All subjects with access to electronic devices and the Internet (e.g. personal computer, smartphone) and fluent in Italian were eligible. Data were collected through a structured self-administered questionnaire created in Google Forms (Google LLC).

Individuals were invited to participate in the survey via social media (Facebook and Whatsapp), and e-mail contacts and the data collection occurred between June and September 2020.

Before starting the questionnaire, participants were informed about the aims of the study and were confident that all data would be used for research purposes only; participants were required to accept the data sharing and privacy policy before taking part in the study. To protect the confidentiality of the participants, their personal information and data were anonymous, according to the provisions of the General Data Protection Regulation (GDPR 679/2016).

### Data collection

The ALT RISCOVID-19/Moli-LOCK questionnaire was constructed by the Department of Epidemiology and Prevention at the IRCCS Neuromed. The questionnaire was divided into modules including questions on socio-demographic characteristics, medical history, COVID-19 related aspects, dietary and lifestyle practices, psychological assessment and sources of information (see online Supplemental Appendix 1).

Changes in UPF consumption were assessed through questions aimed at evaluating modifications possibly occurred during the first Italian lockdown in the intake of nineteen food items grouped according to the NOVA classification system based on the degree of food processing (see online Supplemental Table S1). Briefly, we categorised each food item into one of the following categories according to the extent and purpose of food processing: (1) unprocessed or minimally processed foods (e.g. fruits and vegetables, meat and fish); (2) processed culinary ingredients (e.g. butter, oils); (3) processed foods with salt, sugar or oil (e.g. canned or bottled vegetables and legumes, canned fish) and (4) UPF containing predominantly industrial substances and little or no whole food (e.g. carbonated drinks, processed meat and packaged snacks). For the purpose of the present analyses, we used the fourth NOVA category.

Participants were asked to self-rate their own consumption for each food item in terms of unchanged, increased and decreased intake during confinement as compared to before. Such an approach was chosen to avoid survey fatigue^(19)^ that could have been generated by asking for frequency of consumption before and during pandemic at the same time.

Answers were then used to compute an UPF score by assigning 1 point to increased intake, −1 point to decreased intake while unchanged intakes received 0 points. This score potentially ranges from −19 to 19 with higher values indicating an increase in UPF consumption during confinement. We then divided the population according to degree of changes in UPF as follows: stable UPF intake (UPF score = 0), mild increase (>0 ≤ 2), high increase (>2), mild decrease (≥−2 < 0) and high decrease (<−2).

Lockdown-induced factors included those factors that were likely to be modified by the confinement, namely work type (usual working, home/smart working, work interruption, work reduction, job loss and retired/housewife), income support (no, yes), income reduction (no, yes), physical exercise during confinement (unchanged, increased and decreased), smoking habits (unchanged, increased and decreased), body weight (unchanged, increased and decreased), diagnosis of one or more diseases (no, yes) and any drug use (no, yes).

For the Moli-LOCK participants, we also calculated UPF intake at re-examination (2017–2020) by using dietary data collected through a 188-item FFQ; briefly, we summed the amount consumed (g/d) of each food group included in the fourth category of the NOVA classification (a total of fifteen food groups and three beverages), and then calculated the proportion (%) of UPF in the total weight of food and beverages consumed (g/d) by creating a weight ratio, as previously done in the same cohort^(20)^. Initial UPF intake was then divided into tertiles as low UPF (≤ 8 % of UPF intake on the total food consumed), average (>8 ≤ 12·5 %) and high (>12·5 %).

Also, in both cohorts, we collected data on changes in diet-related behaviours possibly occurred during confinement, such as sources of food supply, number of daily meals, organic food intake and take away food, and for each item we asked whether participants experienced any modification (unchanged, increased and decreased) during lockdown as compared to before.

### Statistical analyses

Data are represented as number and percentage in parentheses (%) for categorical variables or mean (sd) for continuous variables.

Internal consistency for the nineteen UPFs included in the questionnaire was assessed by computing Cronbach’s *α* coefficient (considered satisfactory if higher than or equal to 0·70).

One-way *χ* ^2^ tests were used to assess differences between increased *v*. reduced consumption during lockdown as compared to before.

Regression models adjusted for age groups and sex were used to estimate the association of the UPF score (dependent variable) with demographic and socio-economic correlates; based on multivariable regression analysis (model 2), variables with *P* < 0·10 were included in the multivariable-adjusted regression models used to estimate the association of changes in UPF with lockdown-induced factors and diet-related modifications.

Missing data from categorical variables were assigned a missing indicator. For education, marital status, occupational class, number of cohabitants and living area (< 2 % of missing values) missing values were imputed to the cohort-specific modal value. All analyses were also separately performed for each cohort.

Statistical tests were two-sided, and *P* values < 0·05 were considered to indicate statistical significance.

Data analysis was generated using SAS/STAT software, version 9.4 (SAS Institute Inc.).

## Results

The mean age of our sample of 2992 subjects was 57·9 ± 15·3 years (min-max = 18–91 years), consisted mainly of women (59·6 %), prevalently from Southern Italy (74·4 %).

As compared with the national sample from the ALT RISCOVID-19 survey (*n* 1491; 49·8 %), participants from the Moli-LOCK study (*n* 1501; 50·2 %) were all resident in the Molise region (only two participants moved to Central Italy during follow-up), in Southern Italy, were older and had lower socio-economic status, as reflected by educational level and household income and were more likely to be married/living in couple. Also, they had less prevalence of professional/managerial workers and were more likely to be partly skilled/unskilled workers (Table 1).

**Table 1:** Socio-demographic characteristics of the two cohorts analysed in the current study, Italy 2020

	Both cohorts (*n* 2992)	ALT RISCOVID-19 (*n* 1491)	Moli-LOCK (*n* 1501)
Age (years; mean ± sd)	57·9 ± 15·3	47·9 ± 14·0	67·8 ± 8·5
Men	40·4	36·8	43·9
Geographical areas*			
Northern	17·3	34·7	0·0
Central	6·7	13·3	0·1
Southern and Islands	74·4	48·7	99·9
Living area*			
≥200 000 inhabitants	11·4	22·7	0·1
<200 000 inhabitants	11·9	23·7	0·2
<50 000 inhabitants	54·2	24·1	84·1
Villages/rural areas	22·5	29·4	15·5
Postgraduate education	45·3	70·0	20·8
Household income >40 000 EUR/years	24·0	33·9	14·1
Marital status			
Married/in couple	73·1	63·4	82·8
Single	15·6	27·3	3·9
Divorced	5·4	7·2	3·5
Widower	5·9	2·1	9·8
Occupational class			
Professional/managerial	42·6	58·0	27·3
Skilled non-manual	30·6	22·3	38·8
Skilled manual	5·0	3·1	6·8
Partly skilled/unskilled	6·2	1·8	10·5
Unemployed/unclassified	15·6	14·8	16·5

Values are percentages unless otherwise stated.

* Numbers do not add up to 100 % due to missing data.

UPF included in the questionnaire showed satisfactory internal validity (standardised Cronbach’s coefficient = 0·89). For the majority of UPF here considered, no substantial changes were reported with unchanged consumption varying from 59·3 % (pizza intake) to 90·1 % (breakfast cereals/cereal bars) (Table 2).

**Table 2:** Self-rated changes (%) in the consumption of ultra-processed food during the COVID-19 outbreak confinement in Italy (9 March – 3 May 2020), Italy 2020

	Self-reported changes				
Food items	Unchanged (%)	Increased (%)	Decreased (%)	Overall percent change (%)	*P* value*
Pizza	59·3	31·2	9·6	+21·6	<0·0001
Biscuits	75·8	18·0	6·2	+11·8	<0·0001
Chocolate	73·9	18·6	7·5	+11·1	<0·0001
Bread substitutes	80·3	11·8	7·9	+3·9	<0·0001
Fruit yogurt	87·1	7·7	5·2	+2·5	<0·0001
Breakfast cereals, cereal bars	90·1	4·3	5·6	−1·3	0·017
Sweet packaged snacks	76·8	10·5	12·7	−2·2	0·011
Ready-to-heat potatoes and potato croquettes	83·3	6·8	9·9	−3·1	<0·0001
Packaged bread	81·4	7·7	10·9	−3·2	<0·0001
Fruit drinks (e.g. nectars)	87·9	4·2	7·9	−3·7	<0·0001
Savoury packaged snacks	80·0	7·5	12·5	−5·0	<0·0001
Fish nuggets and sticks	86·5	4·1	9·4	−5·3	<0·0001
Reconstituted meat products	83·9	5·0	11·1	−6·1	<0·0001
Ready-to-heat vegetables	83·6	5·0	11·4	−6·4	<0·0001
Soft drinks	83·0	4·7	12·3	−7·6	<0·0001
Croissants	79·6	6·2	14·2	−8·0	<0·0001
Instant sauces	85·4	3·2	11·4	−8·2	<0·0001
Plant-based meat substitutes	88·6	1·2	10·2	−9·0	<0·0001
Plant-based cheese substitutes (e.g. tofu)	88·8	0·8	10·4	−9·6	<0·0001

* One-way *χ* ^2^ test of increased *v.* decreased consumption.

**Fig. 1:**
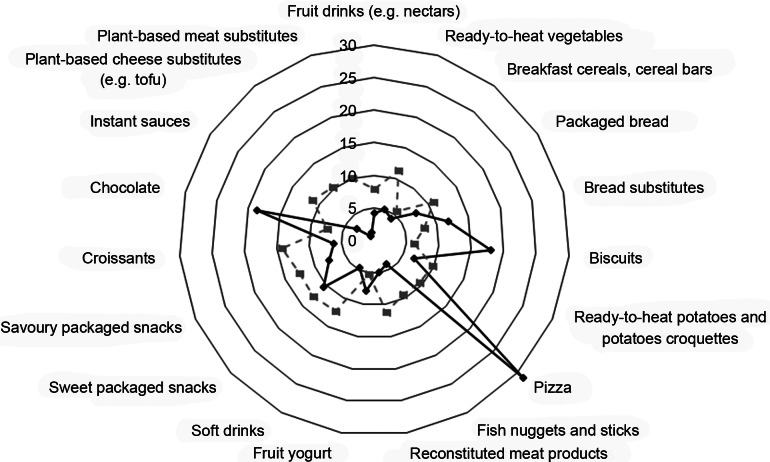
Changes in intake of nineteen ultra-processed foods (UPF) during the Italian lockdown (9 March – 3 May 2020). Radar plots show changes in consumption (increase/decrease) of each food item included in the UPF score along a vertical axis starting in the centre of the circle (0 % change)

Major variations were observed for pizza (31·2 % increase *v*. 9·6 % decrease; *P* value after excluding the unchanged group <0·0001), biscuits (18 % *v*. 6·2 % decrease; *P* value <0·0001), chocolate (18·6 % increase *v*. 7·5 % decrease; *P* value <0·0001) and bread substitutes consumption (11·8 % increase *v*. 7·9 % decrease; *P* value <0·0001), whereas all other foods were decreased (Fig. 1 and Table 2). Analyses by cohort indicated similar results (see online Supplemental Table S2).

**Fig. 2:**
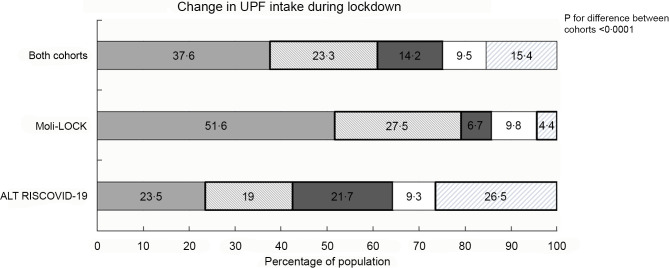
Overall variation in ultra-processed food (UPF) intake during the Italian lockdown (9 March – 3 May 2020) following the COVID-19 pandemic and separately in the ALT RISCOVID-19 and Moli-LOCK study cohorts. 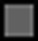, Stable; 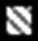, mild increase; 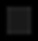, high increase; 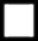, mild decrease; 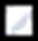, high decrease

The average UPF score was −0·28 (± 4·07, median = 0) indicating that overall the UPF intake at population level during the confinement stayed the same as before. When categorised as UPF change categories, we found that 37·6 % of study participants reported no substantial changes in UPF consumption at individual level, while 37·5 % experienced an increase; among them, 23·3 % reported a mild increase and 14·2 % a high increase in UPF intake (Fig. 2).

Adult/older participants tended to have reduced UPF consumption during lockdown, as compared with younger individuals (*β* = −1·94; 95 % CI −2·72, −1·17 for those aged >75 years *v*. 18–39 years), while men experienced an increase (*β* = 0·40; 95 % CI 0·08, 0·71). Participants from Southern Italian regions reported lower UPF consumption as compared with Northern areas (*β* = −1·32, 95 % CI −1·80, −0·84, respectively), while main socio-economic factors were not associated with UPF changes during lockdown (Table 3).

**Table 3:** Association of demographic and socio-economic factors with self-rated changes in ultra-processed food (UPF) consumption during the Italian lockdown following the COVID-19 pandemic (9 March – 3 May 2020) by means of adjusted regression coefficients (*β*) with 95 % CI and *P*-value, Italy 2020

	%	UPF score mean†	sd †	*β*	95 % CI‡	*P*-value	*β*	95 % CI§	*P*-value
Age groups (years)									
18–39	14·8	−0·36	5·21	Ref.			Ref.		
40–55	23·7	−0·53	5·35	−0·51	−0·99, −0·03	0·039	−0·84	−0·38, −0·31	0·002
56–65	24·4	−0·21	3·76	−0·87	−1·39, −0·34	0·001	−1·35	−1·94, −0·75	<0·0001
66–75	26·8	−0·14	2·73	−1·26	−1·82, −0·70	<0.0001	−1·83	−2·48, −1·19	<0·0001
>75	10·3	−0·09	1·81	−1·38	−2·06, −0·70	<0·0001	−1·94	−2·72, −1·17	<0·0001
Sex									
Women	59·6	−0·48	4·47	Ref.			Ref.		
Men	40·4	0·02	3·37	0·50	0·20, 0·79	0·001	0·40	0·08, 0·71	0·01
Geographical areas*									
Northern	17·3	−0·09	4·88	Ref.			Ref.		
Central	6·7	−0·53	5·47	−0·38	−1·03, 0·27	0·25	−0·38	−1·04, 0·28	0·26
Southern and Islands	74·4	−0·29	3·70	−1·48	−1·93, −1·03	<0·0001	−1·32	−1·80, −0·84	<0·0001
Living area									
≥200 000 inhabitants	11·4	−0·45	4·95	Ref.			Ref.		
<200 000 inhabitants	11·9	−0·51	4·88	−0·09	−0·68, 0·51	0·77	0·38	−0·24, 0·99	0·23
<50 000 inhabitants	54·2	0·07	0·10	−0·58	−1·13, −0·03	0·039	0·004	−0·59, 0·60	0·99
Villages/rural areas	22·5	−0·90	5·20	−1·02	−1·56, −0·49	0·0002	−0·33	−0·92, 0·26	0·28
Educational level									
Up to lower secondary	18·5	−0·04	2·56	Ref.			Ref.		
Upper secondary	36·3	−0·11	3·76	0·17	−0·25, 0·59	0·43	−0·06	−0·53, 0·42	0·81
Postgraduate	45·3	−0·50	4·75	0·32	−0·14, 0·78	0·17	−0·25	−0·83, 0·33	0·41
Household income (EUR/year)									
≤10 000	5·0	−0·41	4·28	Ref.			Ref.		
>10 000 ≤ 25 000	27·4	−0·47	4·28	−0·07	−0·77, 0·62	0·83	−0·27	−0·97, 0·43	0·45
>25 000 ≤ 40 000	29·3	−0·11	3·67	0·28	−0·42, 0·98	0·43	−0·05	−0·78, 0·69	0·90
>40 000 ≤ 60 000	12·7	−0·09	3·88	0·59	−0·18, 1·35	0·13	0·15	−0·67, 0·96	0·73
>60 000	11·3	−0·16	4·29	1·03	0·23, 1·83	0·01	0·44	−0·42, 1·30	0·32
Non respondents	14·3	−0·47	4·34	0·19	−0·56, 0·94	0·62	−0·08	−0·85, 0·68	0·83
Marital status									
Married/in couple	73·1	−0·14	3·74	Ref.			Ref.		
Unmarried	15·6	−0·78	5·33	−0·53	−1·00, −0·06	0·03	−0·29	−0·81, 0·22	0·27
Divorced	5·3	−0·64	5·19	−0·11	−0·76, 0·54	0·74	−0·01	−0·69, 0·67	0·97
Widower	6·0	−0·30	2·80	−0·10	−0·75, 0·55	0·77	0·29	−0·44, 1·01	0·44
Number of cohabitants									
None	10·6	−0·79	4·64	Ref.			Ref.		
1	38·5	−0·12	3·32	0·47	−0·03, 0·97	0·07	0·46	−0·14, 1·06	0·13
2	23·5	0·001	3·98	0·63	0·09, 1·17	0·02	0·69	0·05, 1·32	0·03
>2	27·4	−0·53	4·79	0·16	−0·38, 0·70	0·56	0·21	−0·44, 0·86	0·54
Occupational class									
Professional/managerial	43·5	−0·36	4·39	Ref.			Ref.		
Skilled non-manual	30·9	−0·03	3·71	−0·14	−0·49, 0·22	0·45	−0·12	−0·51, 0·27	0·55
Skilled manual	5·0	−0·25	2·82	−0·55	−1·25, 0·15	0·13	−0·33	−1·10, 0·45	0·41
Partly skilled/unskilled	6·2	−0·11	3·18	−1·50	−1·14, 0·14	0·12	−0·36	−1·06, 0·34	0·32
Unemployed/unclassified	14·5	−0·64	4·48	−0·58	−1·04, −0·13	0·01	−0·42	−0·93, 0·09	0·10

* Numbers do not add up to 100 % due to missing data.

† Unadjusted means.

‡ Multivariable-adjusted linear regression including cohort, age groups and sex.

§ Multivariable-adjusted linear regression including cohort, age groups, sex, geographical area, living area, educational level, household income, marital status, number of cohabitants and occupational class.

Analyses of lockdown-induced correlates (Table 4) revealed that increased physical exercise during lockdown was associated with lower UPF (*β* = −0·90; 95 % CI −1·46, −0·35); those who have increased smoking also tended to consume more UPF as compared with subjects who did not report any change (*β* = 0·75; 95 % CI 0·10, 1·39).

**Table 4:** Association of lockdown-induced factors with self-rated changes in ultra-processed food (UPF) consumption during the Italian lockdown following the COVID-19 pandemic (9 March – 3 May 2020) by means of adjusted regression coefficients (*β*) with 95 % CI and *P*-value, Italy 2020

	%	UPF score mean*	sd *	*β*	95 % CI†	*P*-value
Work type during lockdown						
Usual working	15·4	−0·44	4·64	Ref.		
Smart/home working	23·4	−0·33	4·78	−0·001	−0·47, 0·47	1·00
Work interruption	9·3	−0·97	5·40	−0·53	−1·14, 0·08	0·09
Work reduction	4·7	−0·27	5·63	0·38	−0·37, 1·14	0·32
Job loss	1·0	0·07	5·22	0·56	−0·95, 2·08	0·47
Retired/housewife	42·6	−0·07	2·54	0·03	−0·55, 0·62	0·91
Income support						
No	75·9	−0·23	3·71	Ref.		
Yes	24·1	−0·44	5·04	0·04	−0·33, 0·40	0·84
Income reduction						
No	66·7	−0·04	3·33	Ref.		
Yes	25·8	−0·64	5·26	−0·35	−0·72, 0·01	0·06
Physical exercise during lockdown						
Unchanged	31·5	−0·10	3·40	Ref.		
Increased	9·2	−1·30	4·91	−0·90	−1·46, −0·35	0·001
Decreased	49·6	−0·33	4·49	−0·22	−0·55, 0·11	0·18
Smoking habit during lockdown						
Unchanged	90·1	−0·28	3·94	Ref.		
Increased	5·3	0·07	4·94	0·75	0·10, 1·39	0·023
Decreased	4·6	−0·56	5·39	0·09	−0·60, 0·78	0·81
Diagnosis of chronic diseases during lockdown						
No	95·2	−0·26	4·00	Ref.		
Yes	4·2	−0·26	5·19	0·65	−0·07, 1·37	0·08
Drug use during lockdown						
No	96·1	−0·26	4·00	Ref.		
Yes	3·4	−0·83	5·91	0·04	−0·75, 0·83	0·92
UPF consumption at baseline‡						
Low	33·1	0·17	1·38	Ref.		
Average	33·7	0·51	1·58	0·25	0·06, 0·45	0·01
High	33·2	0·24	1·67	−0·04	−0·24, 0·15	0·68

Numbers do not add up to 100 % due to missing data.

* Unadjusted means.

† Multivariable-adjusted linear regression including cohort, age groups, sex, geographical area, living area, number of cohabitants and occupational class.

‡ Calculated among Moli-LOCK participants only (*n* 1501) and obtained from the multivariable linear regression including age, sex, household income, marital status and occupational class.

Finally, those Moli-LOCK participants (*n* 1501) with an average UPF intake at baseline were more likely to self-rate as increased their UPF intake during confinement (*β* = 0·25; 95 % CI 0·06, 0·45) as compared with low baseline UPF consumers.

### Diet-related behaviours during lockdown

Data indicate an increase in body weight for 37·6 % of respondents and higher number of daily meals for 17·6 % of the sample (Table 5).

**Table 5:** Association of diet-related behaviours with self-rated changes in ultra-processed food (UPF) consumption during the Italian lockdown following the COVID-19 pandemic (9 March – 3 May 2020) by means of adjusted regression coefficients (*β*) with 95 % CI and *P*-value, Italy 2020

	%	UPF score mean*	sd *	*β*	95 % CI†	*P*-value
Body weight						
Unchanged	50·0	−0·63	3·62	Ref.		
Increased	37·6	0·65	4·16	1·33	1·03, 1·64	<0·0001
Decreased	11·4	−1·66	5·48	−1·05	−1·51, −0·59	<0·0001
Take away food						
Unchanged	75·9	−0·13	3·44	Ref.		
Increased	11·6	0·47	5·31	0·78	0·31, 1·25	0·001
Reduced	11·6	−1·75	5·51	−1·24	−1·72, −0·76	<0·0001
Time spent on home food preparation						
Unchanged	49·1	−0·50	3·59	Ref.		
Increased	48·6	−0·002	4·42	0·69	0·39, 0·99	<0·0001
Reduced	1·6	−1·38	5·40	−0·58	−1·72, 0·57	0·32
Number of daily meals						
Unchanged	79·7	−0·57	3·71	Ref.		
Increased	17·6	1·28	5·00	2·15	1·77, 2·52	<0·0001
Reduced	2·4	−1·85	5·04	−1·20	−2·12, −0·27	0·01
Food supplements use						
Unchanged	90·6	−0·21	3·82	Ref.		
Increased	6·8	−0·88	5·48	−0·23	−0·81, 0·36	0·45
Reduced	1·2	−1·00	7·64	−0·38	−1·68, 0·91	0·56
Water intake						
Unchanged	79·6	−0·21	3·49	Ref.		
Increased	17·2	−0·56	5·86	−0·01	−0·39, 0·41	0·95
Reduced	2·8	−0·64	5·67	−0·25	−1·13, 0·64	0·59
Food budget						
Unchanged	55·7	−0·42	3·45	Ref.		
Increased	30·4	0·07	4·90	0·74	0·40, 1·08	<0·0001
Reduced	12·9	−0·48	4·37	0·02	−0·42, 0·46	0·93
Short food supply chain						
Unchanged	56·1	−0·21	3·40	Ref.		
Increased	33·2	−0·40	4·83	0·25	−0·08, 0·57	0·14
Reduced	9·6	−0·09	4·74	0·26	−0·25, 0·76	0·32
Long food supply chain						
Unchanged	54·7	−0·24	3·20	Ref.		
Increased	17·7	−0·01	5·47	0·62	0·22, 1·03	0·003
Reduced	26·5	−0·52	4·55	0·09	−0·26, 0·44	0·61
Organic food						
Unchanged	91·4	−0·14	3·77	Ref.		
Increased	5·2	−1·72	6·77	−1·16	−1·82, −0·50	0·0005
Reduced	1·6	−2·15	5·84	−1·68	−2·83, −0·52	0·005
Local food						
Unchanged	78·9	−0·13	3·69	Ref.		
Increased	14·7	−1·09	5·36	−0·54	−0·96, −0·12	0·01
Reduced	5·0	0·00	4·79	0·11	−0·55, 0·76	0·75
Pre-prepared meals						
Unchanged	82·1	0·18	3·10	Ref.		
Increased	5·2	2·08	4·79	1·84	1·23, 2·46	<0·0001
Reduced	11·6	−4·22	6·39	−4·18	−4·63, −3·73	<0·0001
Long shelf life food						
Unchanged	79·1	−0·05	3·20	Ref.		
Increased	13·8	0·97	5·14	1·20	0·80, 1·60	<0·0001
Reduced	6·2	−5·64	6·30	−5·12	−5·70, −4·54	<0·0001

Numbers do not add up to 100 % due to missing data.

* Unadjusted means.

† Multivariable-adjusted linear regression including cohort, age groups, sex, geographical area, living area, number of cohabitants and occupational class.

**Fig. 3:**
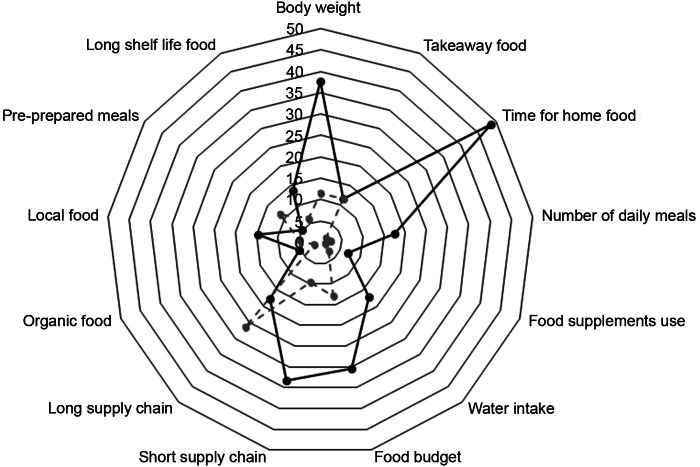
Changes in diet-related behaviours during the Italian lockdown (9 March – 3 May 2020). Radar plots show changes in consumption (increase/decrease) of each diet-related behaviour along a vertical axis starting in the centre of the circle (0 % change)

Increases were found for time spent on home food preparation (48·6 %), short food supply chain (33·2 %), food budget (30·4 %), water intake (17·2 %), organic food (5·2 %), local food (14·7 %), long shelf life products (13·8 %) and dietary supplements (6·8 %); decreases were observed for long supply chain (26·5 %) and pre-prepared meals (11·6 %) (Fig. 3 and Table 5).

Reported reduced body weight during quarantine was associated with lower UPF consumption (*β* = −1·05; 95 % CI −1·51, −0·59) and, consistently, those having increased body weight have also increased UPF (*β* = 1·33; 95 % CI 1·03, 1·64) (Table 5).

A direct association with UPF was found with increased take away (*P* value = 0·001) and number of daily meals (*P* value <0·0001) and increased food budget (*P* value <0·0001). As compared with the unchanged category, UPF was increased among participants with higher long food supply chain shopping (*β* = 0·62; 95 % CI 0·22, 1·03) and decreased among those who have switched to locally grown food (*β* = −0·54; 95 % CI −0·96, −0·12); reduced consumption of pre-prepared meals and long shelf life foods were also associated with lower UPF as compared with unchanged (Table 5).

Analyses by either cohort did not substantially differ from pooled data analysis (see online Supplemental Tables S3–S5).

## Discussion

The present study estimated changes in UPF consumption during the Italian confinement due to the COVID-19 pandemic and contributed to identifying major factors associated with dietary modifications by using data from two population cohorts of adult Italians.

In the analysed sample of 2992 adults, self-rated consumption of UPF seemed to stay the same for the majority of participants, with unchanged response for each food varying from 59·3 to 90·1 %.

When restricted to individuals reporting some variation, analyses revealed significant increases in the intake of pizza, chocolate, biscuits and bread substitutes, and in such our findings are in line with prior works pointing to a raise in sweets and chocolate during lockdown as compared to before^(6–8,21,22)^. On the contrary, intake of many other UPF was found lowered.

To obtain an overall evaluation of dietary changes resulting from weighing all potential responses (i.e. increased, decreased and unchanged intakes), we relied on the use of an UPF score indicating that nearly two-thirds of the population (62·4 %) reported some variations; of them, a total of 37·5 % reported some increase in UPF, varying from mild (23·3 %) to high (14·2 %), while less subjects (24·9 %) reported a decreased UPF consumption.

Our findings are in line with results from a few available surveys analysing changes in diet quality overall during lockdown as compared to before. Longitudinal findings from the web-based NutriNet-Santé cohort study in France on 37 252 adults showed that the lockdown led, in a substantial part of the population, to unhealthy nutritional behaviours^(6)^.

Similarly, data from a convenient sample of nearly 1000 French adults showed an average decrease in nutritional quality of diet which was characterised by a raise in the intake of processed meat, sugary food and sugary beverages during lockdown as compared with before^(23)^.

Others also highlighted that food quality was somehow altered in a health compromising direction during social isolation^(24–26)^.

However, cross-sectional data from Spain showed an improvement in diet quality, as reflected by adherence to a Mediterranean-type diet, during confinement^(27,28)^ and a positive trend towards a Mediterranean diet was also documented in early analyses on an Italian sample^(29)^.

Longitudinal analyses in a sub-cohort of the NutriQuébec study also indicated a slight improvement in the overall diet quality as featured by increased intake of whole grains, seafood and plant proteins^(30)^.

Among socio-demographic factors predisposing to changes in favour of UPF, we found younger age and male subjects both tending to consume more UPF than before the confinement. Also, UPF consumption was unlikely related to major socio-economic factors here analysed, and this was quite surprising in light of the fact that many population-based cohorts^(14,15,20)^, although not all^(12)^, observed a direct relation between high socio-economic status and UPF consumption. However, UPF increase during lockdown seems to be somehow related to higher economic resources being directly associated with increased food budget.

A decrease in UPF consumption was accompanied by other healthy lifestyles during lockdown, such as increased physical activity but also loss of body weight, in line with longitudinal data showing that higher consumption of UPF is associated with a higher risk of multiple indicators of obesity^(31)^. Moreover, studies conducted during the COVID-19 pandemic also showed that unhealthy behaviours tend to cluster, as recently seen in a Spanish population where an unhealthy eating pattern clustered with a less active life during lockdown^(32)^.

In our study, Southern Italian regions were more likely to have decreased UPF possibly because of a stronger Mediterranean diet tradition in these Italian regions^(33)^ which may counterbalance an increase in UPF supply; indeed, it has been proven that respondents closer to healthy diets were less likely to consume UPF^(14)^.

Of note, in the Moli-LOCK study, participants with an already average-high UPF consumption at baseline were more likely to have increased UPF intake during confinement, thus suggesting that those who were already at higher health risk before the COVID-19 pandemic because of unhealthy diets, tended to increase further their long-term risk of disease/mortality.

Individuals who had switched to locally grown food during confinement were also more likely to have reduced their UPF intake; this is consistent with prior evidence showing that UPF are associated with intensive agriculture/livestock, thus being intrinsically unsustainable due to the combination of low-cost ingredients at purchase and increased consumption worldwide^(34)^. Increase of UPF may have negative long-term effect on health as prior data from longitudinal studies showed that habitual consumption of highly processed foods leads to a higher risk of CVD, cancer, diabetes and mortality^(12–15,20)^.

Also, diets rich in processed food, which are typically high in sugars and saturated fats, have been proven to be pro-inflammatory with negative effects also on immune system thus possibly predisposing to higher risk of both chronic and infectious diseases^(16,17)^.

Besides diet modifications, we observed a switch to unhealthy behaviours as reflected by reduced physical exercise for 50 % of respondents and body weight gain for 37·6 % of subjects, in accordance with data on French adults indicating decreased physical activity for 53 % and weight gain for 35 % of subjects^(6)^.

### Strengths and limitations

A major strength of the current study is the use of two population-based cohorts to assess the impact of lockdown on modification of UPF intake. Also, the study has collected data on a number of covariates which limit at least in part confounding and data were collected relatively quickly that is shortly after the end of the first Italian lockdown.

However, our results should be interpreted in light of some limitations. First, the ALT RISCOVID-19 is a web-based survey on a convenience sample with potential selection bias and self-reported information that may lead to misreporting. However, our analyses also rely on data collected within the Moli-LOCK study which includes participants from a population-based prospective cohort who were phone interviewed and this limits misreporting. Indeed, main findings from pooled analysis were substantially confirmed by separate analyses by cohort, supporting the actual validity of the observed associations. Both cohorts used data collected retrospectively thus recall bias cannot be excluded. Change in UPF consumption was self-rated rather than assessed more objectively through a simultaneous administration of dietary questionnaires before and during lockdown; however, our approach is the same used by the majority of studies published to date on this topic^(7,21,22,29,35–37)^ and is likely to be a pragmatic solution to conducting research on a large sample in relation to an unexpected event^(36,37)^.

Also, from a methodological point of view, either studies using validated tools (e.g. FFQ) to assess diet before and during pandemic simultaneously or those relying on self-rated changes in terms of increased/decreased/unchanged consumption, share the same limitations, mostly represented by flawed recall. Moreover, it is noteworthy that, although able to estimate quantitative changes, the double administration of validated dietary scales could be still deficient since it might lack specificity to the current pandemic state.

## Conclusions

During the first Italian lockdown from 9 March to 3 May 2020, UPF consumption seemed to stay the same for the majority of participants, although a sub-group of the surveyed population (37·5 %) tended to switch to unhealthy eating as reflected by increased UPF intake as compared with before.

In view of possible future containment measures to tackle new COVID-19 outbreaks, it is crucial that unhealthy lifestyles adopted during the lockdowns are immediately targeted in order to avoid maintenance of unfavourable behaviours that may increase the nutrition-related burden of disease^(38)^. Future longitudinal studies are warranted to examine the long-term effects of the lockdown on dietary habits possibly relying on more accurate dietary assessments.

## Supplementary material

For supplementary material accompanying this paper visit http://dx.doi.org/10.1017/S1368980021000999. click here to view supplementary material

## References

[1] World Health Organization, virtual press conference on COVID-19 – 11 March 2020; available at https://www.who.int/docs/default-source/coronaviruse/transcripts/who-audio-emergencies-coronavirus-press-conference-full-and-final-11mar2020.pdf?sfvrsn=cb432bb3_2 (accessed October 2020).

[2] John Hopkins University, 2021; available at https://coronavirus.jhu.edu/map.html (accessed January 2021).

[3] Italian Ministry of Health (2020) Covid-19, how to follow an appropriate and healthy lifestyle when staying at home; available at http://www.salute.gov.it/portale/nuovocoronavirus/dettaglioNotizieNuovoCoronavirus.jsp?lingua=italiano&menu=notizie&p=dalministero&id=4421 (accessed October 2020).

[4] Mattioli AV , Ballerini Puviani M , Nasi M et al. (2020) COVID-19 pandemic: the effects of quarantine on cardiovascular risk. Eur J Clin Nutr 74, 852–855. 32371988 10.1038/s41430-020-0646-z PMC7199203

[5] Muscogiuri G , Barrea L , Savastano S et al. (2020) Nutritional recommendations for CoVID-19 quarantine. Eur J Clin Nutr 74, 850–851. 32286533 10.1038/s41430-020-0635-2 PMC7155155

[6] Deschasaux-Tanguy M , Druesne-Pecollo N , Esseddik Y et al. (2020) Diet and physical activity during the COVID-19 lockdown period (March–May 2020): results from the French NutriNet-sante cohort study. MedRxiv. Published online: 5 June 2020. doi: 10.1101/2020.06.04.20121855.

[7] Błaszczyk-Bębenek E , Jagielski P , Bolesławska I et al. (2020) Nutrition behaviors in polish adults before and during COVID-19 lockdown. Nutrients 12, 3084. 10.3390/nu12103084 PMC7601522 33050404

[8] Giacalone D , Frøst MB & Rodríguez-Pérez C (2020) Reported changes in dietary habits during the COVID-19 lockdown in the Danish population: the Danish COVIDiet study. Front Nutr 7, 592112. 33364250 10.3389/fnut.2020.592112 PMC7752855

[9] Louzada ML , Baraldi LG , Steele EM et al. (2015) Consumption of ultra-processed foods and obesity in Brazilian adolescents and adults. Prev Med 81, 9–15. 26231112 10.1016/j.ypmed.2015.07.018

[10] Monteiro CA , Moubarac JC , Cannon G et al. (2013) Ultra-processed products are becoming dominant in the global food system. Obes Rev 14, 21–28. 24102801 10.1111/obr.12107

[11] Monteiro CA , Cannon G , Moubarac JC et al. (2018) The UN decade of nutrition, the NOVA food classification and the trouble with ultra-processing. Public Health Nutr 21, 5–17. 28322183 10.1017/S1368980017000234 PMC10261019

[12] Srour B , Fezeu LK , Kesse-Guyot E et al. (2019) Ultra-processed food intake and risk of cardiovascular disease: prospective cohort study (NutriNet-Santé). BMJ 29, l1451. 10.1136/bmj.l1451 PMC6538975 31142457

[13] Pagliai G , Dinu M , Madarena MP et al. (2021) Consumption of ultra-processed foods and health status: a systematic review and meta-analysis. Br J Nutr 125, 308–318. 32792031 10.1017/S0007114520002688 PMC7844609

[14] Rico-Campà A , Martínez-González MA , Alvarez-Alvarez I et al. (2019) Association between consumption of ultra-processed foods and all cause mortality: SUN prospective cohort study. BMJ 365, l1949. 31142450 10.1136/bmj.l1949 PMC6538973

[15] Blanco-Rojo R , Sandoval-Insausti H , López-Garcia E et al. (2019) Consumption of ultra-processed foods and mortality: a national prospective cohort in Spain. Mayo Clin Proc 94, 2178–2188. 31623843 10.1016/j.mayocp.2019.03.035

[16] Han YY , Jerschow E , Forno E et al. (2020) Dietary patterns, asthma, and lung function in the Hispanic community health study/study of Latinos. Ann Am Thorac Soc 17, 293–301. 31689128 10.1513/AnnalsATS.201908-629OC PMC7044698

[17] Morais AHA , Aquino JS , da Silva-Maia JK et al. (2020) Nutritional status, diet and viral respiratory infections: perspectives for severe acute respiratory syndrome coronavirus 2. Br J Nutr 26, 1–12. 10.1017/S0007114520003311 PMC7542326 32843118

[18] Iacoviello L , Bonanni A , Costanzo S et al. (2007) The MOLI-SANI Project, a randomized, prospective cohort study in the Molise region in Italy; design, rationale and objectives. Italian J Public Health 4, 110–118.

[19] O’Reilly-Shah VN (2017) Factors influencing healthcare provider respondent fatigue answering a globally administered in-app survey. PeerJ 12, e3785. 10.7717/peerj.3785 PMC5600176 28924502

[20] Bonaccio M , Di Castelnuovo A , Costanzo S et al. (2020) Ultra-processed food consumption is associated with increased risk of all-cause and cardiovascular mortality in the Moli-sani Study. Am J Clin Nutr 113, 446–455. 10.1093/ajcn/nqaa299 33333551

[21] Vandevijvere S , De Ridder K , Drieskens S et al. (2021) Food insecurity and its association with changes in nutritional habits among adults during the COVID-19 confinement measures in Belgium. Public Health Nutr 24, 950–956. 33292888 10.1017/S1368980020005005 PMC7804079

[22] Scarmozzino F & Visioli F (2020) Covid-19 and the subsequent lockdown modified dietary habits of almost half the population in an Italian sample. Foods 9, 675. 10.3390/foods9050675 PMC7278864 32466106

[23] Marty L , de Lauzon-Guillain B , Labesse M et al. (2020) Food choice motives and the nutritional quality of diet during the COVID-19 lockdown in France. Appetite 14, 105005. 10.1016/j.appet.2020.105005 PMC7558232 33068666

[24] Alhusseini N & Alqahtani A (2020) COVID-19 pandemic’s impact on eating habits in Saudi Arabia. J Public Health Res 9, 1868. 33024727 10.4081/jphr.2020.1868 PMC7512943

[25] Ammar A , Brach M , Trabelsi K et al. (2020) Effects of COVID-19 home confinement on eating behaviour and physical activity: results of the ECLB-COVID19 international online survey. Nutrients 12, 1583. 10.3390/nu12061583 PMC7352706 32481594

[26] Malta DC , Szwarcwald CL , Barros MBA et al. (2020) The COVID-19 pandemic and changes in adult Brazilian lifestyles: a cross-sectional study, 2020. Epidemiol Serv Saude 2020, e2020407. 10.1590/S1679-49742020000400026 32997069

[27] Rodríguez-Pérez C , Molina-Montes E , Verardo V et al. (2020) Changes in dietary behaviours during the COVID-19 outbreak confinement in the Spanish COVIDiet study. Nutrients 12, 1730. 10.3390/nu12061730 PMC7353108 32531892

[28] Sánchez-Sánchez E , Ramírez-Vargas G , Avellaneda-López Y et al. (2020) Eating habits and physical activity of the Spanish population during the COVID-19 pandemic period. Nutrients 12, 2826. 10.3390/nu12092826 PMC7551353 32942695

[29] Di Renzo L , Gualtieri P , Pivari F et al. (2020) Eating habits and lifestyle changes during COVID-19 lockdown: an Italian survey. J Transl Med 18, 229. 32513197 10.1186/s12967-020-02399-5 PMC7278251

[30] Lamarche B , Brassard D , Lapointe A et al. (2021) Changes in diet quality and food security among adults during the COVID-19-related early lockdown: results from NutriQuébec. Am J Clin Nutr 5, nqaa363. 10.1093/ajcn/nqaa363 PMC7799255 33398347

[31] Rauber F , Chang K , Vamos EP , da Costa Louzada ML et al. (2021) Ultra-processed food consumption and risk of obesity: a prospective cohort study of UK Biobank. Eur J Nutr 60, 2169–2180. 33070213 10.1007/s00394-020-02367-1 PMC8137628

[32] Pérez-Rodrigo C , Gianzo Citores M , Hervás Bárbara G et al. (2021) Patterns of change in dietary habits and physical activity during lockdown in Spain due to the COVID-19 pandemic. Nutrients 13, 300. 33494314 10.3390/nu13020300 PMC7911477

[33] Ruggiero E , Di Castelnuovo A , Costanzo S et al. (2019) Socioeconomic and psychosocial determinants of adherence to the Mediterranean diet in a general adult Italian population. Eur J Public Health 29, 328–335. 30020486 10.1093/eurpub/cky127

[34] Fardet A & Rock E (2020) Ultra-processed foods and food system sustainability: what are the links? Sustainability 12, 6280.

[35] Bin Zarah A , Enriquez-Marulanda J & Andrade JM (2020) Relationship between dietary habits, food attitudes and food security status among adults living within the United States 3 months post-mandated quarantine: a cross-sectional study. Nutrients 12, 3468. 10.3390/nu12113468 PMC7697798 33198215

[36] Coulthard H , Sharps M , Cunliffe L et al. (2021) Eating in the lockdown during the Covid 19 pandemic; self-reported changes in eating behaviour, and associations with BMI, eating style, coping and health anxiety. Appetite 161, 105082. 33476651 10.1016/j.appet.2020.105082 PMC7976455

[37] Phillipou A , Meyer D , Neill E et al. (2020) Eating and exercise behaviors in eating disorders and the general population during the COVID-19 pandemic in Australia: initial results from the COLLATE project. Int J Eat Disord 53, 1158–1165. 32476163 10.1002/eat.23317 PMC7300745

[38] Dai H , Much AA , Maor E et al. (2020) Global, regional, and national burden of ischemic heart disease and its attributable risk factors, 1990–2017: results from the global Burden of Disease Study 2017. Eur Heart J Qual Care Clin Outcomes qcaa076. doi: 10.1093/ehjqcco/qcaa076. 10.1093/ehjqcco/qcaa076 PMC8728029 33017008

