# Supplementary material for: Changes in ultra-processed food consumption during the first Italian lockdown following the COVID-19 pandemic and major correlates: results from two population-based cohorts
Source: Public Health Nutr. 2021 Mar 5:1–11. doi: 10.1017/S1368980021000999 (PMC8207556; doi:10.1017/S1368980021000999)
Supplement: Supplementary file 1 [file S1368980021000999sup001.docx]

**Supplementary Figure 1.** Flowchart for selection of study participants from the Moli-LOCK cohort Study

**
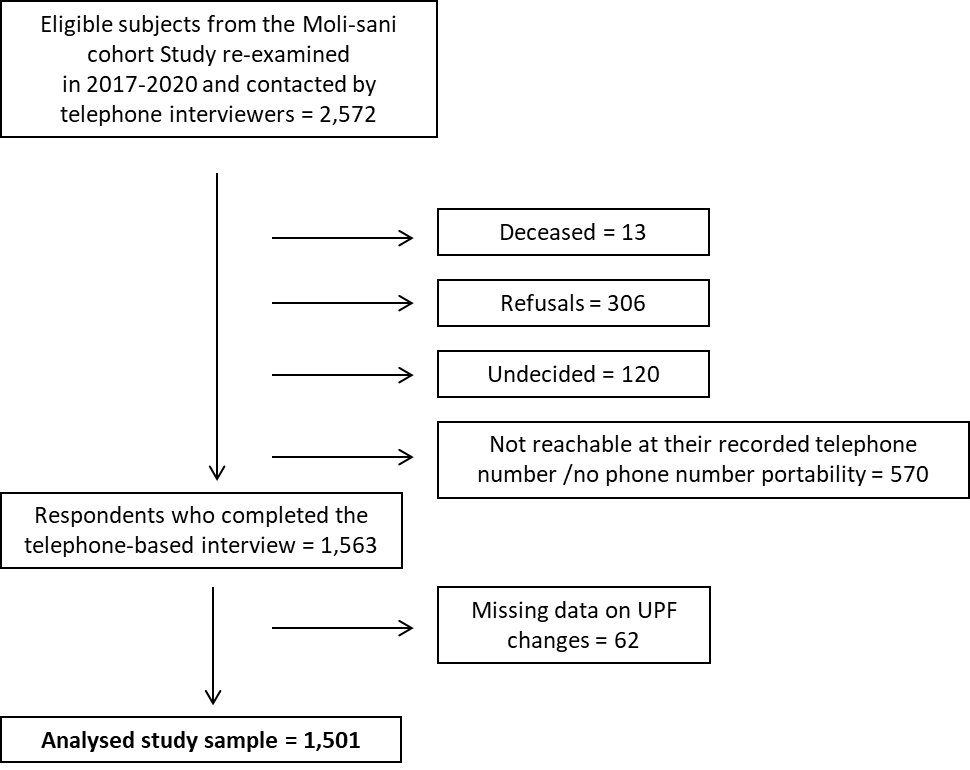
**

**Supplementary Appendix 1.** ALT RISCOVID-19/Moli-LOCK questionnaire

| **Questions** | **Answers** |
| --- | --- |
|  |  |
| **Personal information** |  |
| Birth year | Birth year |
| Gender | Female/Male/Other |
| Ethnicity | Europe  Asia  North America  South/Central America  Africa  Oceania  Other  Refused/I do not know |
| Place of residence | ZIP code or city name |
|  |  |
| **Socioeconomic factors** |  |
| Educational level | None  Primary school  Lower secondary school  Upper secondary  University degree  Postgraduate, Master’s degree, Doctorate  Refused/I do not know |
| Type of employment contract | Full-time employed  Part-time employed  Self-employed  Student  Retiree  Unemployed  Citizen’s income  Unemployment benefits  Housewife  Disability benefits  Other  Refused/I do not know |
| Main employment in the lifetime | Armed forces occupations  Managers  Professionals  Technicians and associate professionals  Clerical support workers  Service and sales workers  Skilled agricultural, forestry and fishery workers  Craft and related trades workers  Plant and machine operators and assemblers  Elementary occupations  Refused/I do not know |
| Are you a healthcare professional? | No, I’m not  GP (general practitioner)  Hospital-based general physician  Clinical physician  Nurse  Volunteer EMT  Intermediate care technician  Pharmacist  Other  Refused/I do not know |
| Household income in 2019 (EUR/y) | ≤10.000  >10.000≤25.000  >25.000≤40.000  >40.000≤60.000  >60.000≤100.000  >100.000  Refused/I do not know |
| Housing tenure | Rent  1 dwelling ownership  > 1 dwelling ownership  Refused/I do not know |
| Number of rooms in the house (bathrooms and additional rooms excluded) | 1  2  3  > 3  Refused/I do not know |
| Number of people living in the house (excluding respondent) | None  1  2  more than 2  Refused/I do not know |
| Marital status | Unmarried  Married/in couple  Divorced/separated  Widower  Refused/I do not know |
| Place of living | City centre > 200.000 inhabitants  City suburb > 200.000 inhabitants  City centre < 200.000 inhabitants  City outskirts < 200.000 inhabitants  City < 50.000 inhabitants  Small city/village < 10.000 inhabitants  Rural area  Refused/I do not know |
| The road traffic where you live is | High  Moderate  Low  Refused/I do not know |
| Do you live with someone older than 65 y? | Yes/No/Refused/I do not know |
| Do you live with someone younger than 12 y? | Yes/No/Refused/I do not know |
| Do you live with a disabled person? | Yes/No/Refused/I do not know |
|  |  |
| **Health information** |  |
| Have you been diagnosed with some of these diseases immediately before Phase 1 of lockdown? (multiple choice) | Lung disease  Hearth disease  Ischemic/haemorrhagic stroke  Hypertension  Diabetes  Kidney disease  Immune system disease  Cancer  Liver disease  Depression  Anxiety  Parkinson’s disease  Alzheimer’s disease or dementia  Multiple sclerosis  None  Refused/I do not know |
| Are you disabled? | Yes/No/Refused/I do not know |
| Did you regularly take some of these drugs immediately before Phase 1 of lockdown? (multiple choice) | Aspirin  Hearth disease drugs  Blood pressure medications  Cholesterol medications  Diabetes drugs  Anti-cancer drugs  Corticosteroids  Thyroid medications  Anti-inflammatory drugs  Anxiolytics  Antidepressants  Immunomodulators  None  Refused/I do not know |
| Did you get flu vaccination last autumn (2019)? | Yes/No/Refused/I do not know |
| Did you get anti pneumococcal vaccine last autumn (2019)? | Yes/No/Refused/I do not know |
|  |  |
| **COVID-19** |  |
| From February 1 till now, did you get in touch with someone who contracted COVID-19 disease or was identified as positive by tests? | No, I did not  Yes, in the workplace  Yes, not in the workplace  Refused/I do not know |
| Are you worried about getting infected by COVID-19? | Not at all  A little bit  Quite enough  Extremely |
| Are you worried your relatives could get be infected by COVID-19? | Not at all  A little bit  Quite enough  Extremely |
| Since February 1 till now, did you have any of these symptoms? (multiple choice) | Temperature higher than 37,5 °C for 3 consecutive days  Cough  Sore throat/flu  Headache  Muscle/bone/joint pain  Loss of taste (ageusia)/loss of smell  Respiratory distress  Gastrointestinal symptoms (diarrhoea, nausea, vomit)  Conjunctivitis  Pneumonia  None  Refused/I do not know |
| Did you have a swab test for COVID-19? | No, I did not  Yes, it tested positive  Yes, it tested negative  Yes, it tested unclear  Refused/I do not know |
| Did you repeat the swab test? | No, I did not  Yes, it tested positive  Yes, it tested negative  Yes, it tested unclear  Refused/I do not know |
| Did you have a serology test for COVID-19? | No, I did not  Yes, it resulted positive  Yes, it resulted negative  Yes, it resulted unclear  Refused/I do not know |
| Were you diagnosed with COVID-19? | No, I was not  Yes, I was treated at home  Yes, I was treated at the hospital  Yes, I was admitted into intensive care unit  Yes, I was intubated  Refused/I do not know |
| If you got infected by COVID-19, have you had any of the following persistent neurological symptoms even after been declared healed? (multiple choice) | Loss of taste  Loss of smell  Hearing loss  Tremors or movement disorders  Seizures  None  Refused/I do not know |
| **Impact of lockdown on daily life** |  |
| What about your working activity during Phase 1 (March 9 to May 3, 2020)? | I went to work as usual  I worked in smart working at home  I suspended my work  I reduced my work  I was out of work (dismissal)  Refused/I do not know |
| What about your working activity after Phase 1? | I work in my workplace as usual  I am doing smart working at home  I suspended my work  I reduced my work  I was out of work (dismissal)  Refused/I do not know |
| Did your family have income support measures during Phase 1? (multiple choice) | Unemployment benefit  600 € bonus  Baby-sitting bonus  Parental leave bonus  None  Other  Refused/I do not know |
| How much did the monthly household income was reduced during Phase 1? | No reduction  **<10%**  **10-20%**  **21-30%**  **31-50%**  **51-70%**  **>70%**  **Refused/I do not know** |
| **Have you been diagnosed with any of the following diseases during Phase 1?** (multiple choice) | Lung disease  Hearth disease  Ischemic/ haemorrhagic stroke  Hypertension  Diabetes  Kidney disease  Immune system disease  Cancer  Liver disease  Depression  Anxiety  Parkinson’s disease  Alzheimer’s disease or dementia  Multiple sclerosis  None  Refused/I do not know |
| **Did you give up medical checks or tests during Phase1?** | **No, I** did not  **Yes, I gave up**  **Yes, my medical check has been cancelled/delayed**  **Refused/I do not know** |
| **Did you quit taking any of the following drugs during Phase 1? (multiple choice)** | **Aspirin**  **Hearth disease drugs**  **Blood pressure medications**  **Cholesterol medications**  **Diabetes drugs**  **Anti-cancer drugs**  **Corticosteroids**  **Thyroid medications**  **Anti-inflammatory drugs**  Anxiolytics  Antidepressants  **Immunomodulators**  **None**  **Refused/I do not know** |
| **During Phase 1 did you start using**  **(multiple choice)** | **Sleeping pills**  **Anxiolytics**  **Antidepressants**  **None**  **Refused/I do not know** |
| **When did you use to wear the face mask during Phase 1?** | **Never used**  **Only to go out for a walk/ play sports**  **Only to access the stores**  **Each time I went out**  **Every time, even at home**  **Refused/I do not know** |
| **When do you use to wear the face mask after Phase 1?** | **Never**  **Only to go out for a walk/ play sports**  **Only to access the stores**  **Each time I go out**  **Every time, even at home**  **Refused/I do not know** |
| **Lifestyles** |  |
| **Did you smoke before Phase 1?** | **I did not use to smoke/I have never smoked**  **I was a former smoker**  **I used to smoke less than 10 cigarettes per day**  **I used to smoke between 10 to 20 cigarettes per day**  **I used to smoke more than 20 cigarettes per day**  **Refused/I do not know** |
| **How did you change your smoking habit during Phase 1?** | **I smoked as before**  **I smoked more than before**  **I smoked less than before**  **I started smoking**  **I quitted smoking**  **Refused/I do not know** |
| **How was your physical activity before Phase 1?** | **Never had or practiced less than 30 min per week**  **Between 30 min and 2 hours and 30 per week**  **More than 2 hours and 30 per week**  **Refused/I do not know** |
| **Your physical activity during Phase 1 was** | **Increased**  **Decreased**  Unchanged  Refused/I do not know |
| How many times per week did you go out during Phase 1? | Never  Once a week  2-3 times a week  4-5 times a week  6 or more times a week  Refused/I do not know |
| Did you get public transport during Phase 1? | Not at all  Yes, 1 to 3 times per week  Yes, 4 to 6 times per week  Yes, 7 or more times per week  Refused/I do not know |
| **Sources of information** |  |
| During Phase 1 you followed general news | Less than usual  More than usual  As usual  Refused/I do not know |
| Were you interested in specific news about COVID-19? | Not at all  A little bit  Quite enough  Extremely  Refused/I do not know |
| How did you find out news during Phase 1? (multiple choice) | Television  Radio  Newspapers  Television, radio or newspapers websites  Government websites  Social networks  Friends  GP  Refused/I do not know |
| Who did you trust the most about COVID-19 emergency? | Scientists  Doctors  Journalists  Political authorities  News on social networks  Friends  Refused/I do not know |
| You think that during Phase 1 news has been | Substantially reliable  Confusing  Too conflicting with each other  Manipulated for economic or political interests  Refused/I do not know |
|  |  |
| **Diet-related factors** |  |
| *During Phase 1 lockdown:* |  |
| Your body weight | Increased/Decreased/Unchanged/Refused/I do not know |
| Take-away food consumption | Increased/Decreased/Unchanged/Refused/I do not know |
| Time spent on home  food preparation (e.g. bread, desserts, pasta, pizza) i | Increased/Decreased/Unchanged/Refused/I do not know |
| The number of meals per day (including snacks) | Increased/Decreased/Unchanged/Refused/I do not know |
| Food supplements (e.g. vitamins) | Increased/Decreased/Unchanged/Refused/I do not know |
| Water consumption | Increased/Decreased/Unchanged/Refused/I do not know |
| Food budget | Increased/Decreased/Unchanged/Refused/I do not know |
| Food shopping in short supply chain | Increased/Decreased/Unchanged/Refused/I do not know |
| Food shopping in long supply chain | Increased/Decreased/Unchanged/Refused/I do not know |
| Organic food consumption | Increased/Decreased/Unchanged/Refused/I do not know |
| Local food consumption | Increased/Decreased/Unchanged/Refused/I do not know |
| Pre-prepared meals consumption | Increased/Decreased/Unchanged/Refused/I do not know |
| Long shelf life food consumption | Increased/Decreased/Unchanged/Refused/I do not know |
|  |  |
| **Diet** |  |
| Fresh fruit | Increased/Decreased/Unchanged/Refused/I do not know |
| Nuts and seeds |  |
| Fruit juices/nectars (with added sugars) |  |
| Fresh vegetables |  |
| Packaged vegetables |  |
| Ready-to-heat vegetables |  |
| Fresh/dried legumes |  |
| Canned legumes |  |
| Cereals (bread, pasta, rice, couscous) |  |
| Wholegrain cereals (bread, pasta, rice, couscous) |  |
| Breakfasts cereals and bars |  |
| Packaged bread |  |
| Bread alternatives (crackers, taralli, breadsticks, frisella, rusks) |  |
| Biscuits |  |
| Potatoes |  |
| Ready-to-heat chips and potato croquettes |  |
| Pizza/focaccia |  |
| Fresh/frozen fish  (no added ingredients) |  |
| Dried, smoked or salted fish (e.g. stock fish, smoked salmon) |  |
| Canned fish |  |
| Fish sticks nuggets (with added ingredients) |  |
| Red meat |  |
| White meat (chicken, turkey, rabbit) |  |
| Salted, dried, cured, or smoked meats |  |
| Reconstituted meat products |  |
| Milk/plain yoghurt |  |
| Fruit yoghurt |  |
| Soft cheese (e.g. mozzarella) |  |
| Hard cheese (e.g. pecorino) |  |
| Eggs |  |
| Olive oil |  |
| Butter, margarine, sour cream |  |
| Wine/beer/alcoholic beverages |  |
| Soft drinks  (e.g. cold tea, cola, chinotto, orange soda, soda water) |  |
| Coffee |  |
| Sweet packaged snacks |  |
| Savoury packaged snacks |  |
| Croissants/pastries/brioche |  |
| Chocolate |  |
| Instant sauces (e.g. Pesto) |  |
| Plant-based cheese substitutes (e.g. Tofu) |  |
| Plant-based meat substitutes (e.g. veggie burger) |  |
|  |  |
| **Psychological aspects** |  |
|  |  |
| **Patient’s Health Questionnaire (PHQ-9)** |  |
| *During the Phase 1 lockdown, how often have you been bothered by the following?* |  |
| 1. Little interest or pleasure in doing things? | never, several days, more than half the days, nearly every day |
| 1. Feeling down, depressed, or hopeless? |  |
| 1. Trouble falling or staying asleep, or sleeping too much? |  |
| 1. Feeling tired or having little energy? |  |
| 1. Poor appetite or overeating? |  |
| 1. Feeling bad about yourself - or that you are a failure or have let yourself or your family down? |  |
| 1. Trouble concentrating on things, such as reading the newspaper or watching television? |  |
| 1. Moving or speaking so slowly that other people could have noticed?   Or the opposite - being so fidgety or restless that you have been moving around a lot more than usual? |  |
| 1. Thoughts that you would be better off dead, or of hurting yourself in some way? |  |
|  |  |
| **Generalized Anxiety Disorder 7-Item Scale (GAD-7)** |  |
| *During Phase 1, how often did each of the following bother you?* |  |
| 1. Feeling nervous, anxious, or on edge | not at all, several days, more than half the days, nearly every day |
| 1. Not being able to stop or control worrying |  |
| 1. Worrying too much about different things |  |
| 1. Trouble relaxing |  |
| 1. Being so restless that it's hard to sit still |  |
| 1. Becoming easily annoyed or irritable |  |
| 1. Feeling afraid as if something awful might happen |  |
|  |  |
| **Social support** |  |
| *During PHASE 1, how much did you count on the support / help of these people?* |  |
| 1. Family members or other relatives | never, sometimes, often, all the time |
| 1. Friends / acquaintances / neighbours |  |
| 1. Other people free of charge (e.g. volunteers from associations) |  |
| 1. Other paid persons (e.g. caregiver, housekeeper) |  |
| 1. Professionals (e.g. psychologists, doctors) |  |
| 1. During PHASE 1, did you feel you were supporting / helping someone? | Not at all, a little, moderately, much, very much |
|  |  |
| **Perceived Stress Scale 4 (PSS-4)** |  |
| *During Phase 1, how often* |  |
| 1. Have you felt that you were unable to control the important things in your life? | never, almost never, sometimes, fairly often, very often |
| 1. Have you felt confident about your ability to handle your personal problems? |  |
| 1. Have you felt that things were going your way? |  |
| 1. Have you felt difficulties were piling up so high that you could not overcome them? |  |
|  |  |
| **Screening Questionnaire for Disaster Mental Health (SQD)** |  |
| *Have you experienced any of the symptoms listed below in relation to the COVID-19 pandemic IN THE LAST MONTH?* |  |
| 1. Have you noticed any changes in your appetite? | Yes, No |
| 1. Do you feel that you are easily tired and / or tired all the time? |  |
| 1. Do you have trouble falling asleep or sleeping through the night? |  |
| 1. Do you have nightmares about the event? |  |
| 1. Do you feel depressed? |  |
| 1. Do you feel irritable? |  |
| 1. Do you feel that you are hypersensitive to small noises or tremors? |  |
| 1. Do you avoid places, people, topics related to the event? |  |
| 1. Do you think about the event when you do not want to? |  |
| 1. Do you have trouble enjoying things you used to enjoy? |  |
| 1. Do you get upset when something reminds you of the event? |  |
| 1. Do you notice that you are making an effort to try not to think about the event, or are trying to forget it? |  |
|  |  |
| **‘Hut syndrome’** |  |
| 1. How much you feel worried by the fact that you are gradually returning to your habits and therefore being able to leave the house (not only for food supply)? | not at all, slightly, moderately, enough, a lot |
| 1. How much you feel worried by the fact that your family is gradually returning to its habits and therefore being able to leave the house (not just for food supply)? | not at all, slightly, moderately, enough, a lot |
| 1. What worries you most about returning to work | Public transport, meeting colleagues, meeting the public/customers, share common areas for lunch/coffee break, share toilets, I don't have to go back to work/work from home, I'm not worried, don't know/don't want to answer |
| 1. Compared to the period before the lockdown PHASE 1, now you feel: | More comfortable at home, more comfortable away from home, as usual |

**Supplementary Table 1.** Food items grouping according to the NOVA classification

| **Group 1.** Unprocessed or minimally processed foods | Fresh fruit, nuts, fresh vegetables, dried legumes, cereals (e.g. pasta, bread, rice), fresh fish, red meat, poultry, egg, potatoes, milk and plain yogurt, coffee. |
| --- | --- |
| **Groups 2 and 3.** Culinary ingredients and processed foods | Olive oil, butter/lard/sour cream, wine/beer, salted/dried/ cured or smoked fish, canned fish, canned legumes, soft cheese, hard cheese, salted, cured, or smoked meats. |
| **Group 4.** Ultra-processed foods | Fruit drinks, packaged bread, bread alternatives, breakfast cereals and cereals bars, biscuits, ready-to-heat potatoes and potato croquettes, pizza, ready-to-heat vegetables, fish nuggets, reconstituted meat products, fruit yogurts, soft drinks, sweet packaged snacks, savoury packaged snacks, croissants, chocolate, instant sauces, plant-based cheese substitutes, plant-based meat substitutes. |

**Supplementary Table 2.** Self-rated changes (%) in consumption of ultra-processed food during the COVID-19 outbreak confinement in Italy (March 9 – May 3, 2020) in the ALT RISCOVID-19 and Moli-LOCK study cohorts, Italy 2020

|  | **ALT RISCOVID-19 (n=1,491; 49.8%)** | | | | **Moli-LOCK Study (n=1,501; 50.2%)** | | | |
| --- | --- | --- | --- | --- | --- | --- | --- | --- |
| **Food items** | Unchanged (%) | Increased (%) | Decreased (%) | Overall percent  reduction (%) | Unchanged (%) | Increased (%) | Decreased (%) | Overall percent reduction (%) |
| Pizza | 49.0 | 38.8 | 12.1 | 26.7 | 69.4 | 23.6 | 7.0 | 16.6 |
| Chocolate | 59.8 | 27.8 | 12.5 | 15.3 | 87.9 | 9.5 | 2.6 | 6.9 |
| Biscuits | 70.4 | 19.1 | 10.5 | 8.6 | 81.3 | 16.9 | 1.9 | 15.0 |
| Fruit yogurt | 76.2 | 13.9 | 9.9 | 4.0 | 97.9 | 1.7 | 0.4 | 1.3 |
| Bread substitutes | 66.8 | 18.4 | 14.8 | 3.6 | 93.8 | 5.2 | 1.0 | 4.2 |
| Sweet packaged snacks | 63.6 | 17.2 | 19.2 | -2.0 | 89.8 | 3.9 | 6.3 | -2.4 |
| Breakfast cereals, cereal bars | 80.9 | 8.2 | 10.9 | -2.7 | 99.3 | 0.3 | 0.3 | 0.0 |
| Savoury packaged snacks | 66.8 | 14.2 | 19.0 | -4.8 | 93.2 | 0.8 | 6.0 | -5.2 |
| Ready-to-heat potatoes and potato croquettes | 68.5 | 12.9 | 18.6 | -5.7 | 97.9 | 0.8 | 1.3 | -0.5 |
| Packaged bread | 64.6 | 14.2 | 21.3 | -7.1 | 98.1 | 1.3 | 0.6 | 0.7 |
| Fruit drinks (e.g. nectars) | 77.0 | 7.8 | 15.2 | -7.4 | 98.8 | 0.60 | 0.60 | 0.0 |
| Reconstituted meat products | 72.2 | 8.5 | 19.3 | -10.8 | 95.5 | 1.5 | 3.0 | -1.5 |
| Fish nuggets and sticks | 74.9 | 7.0 | 18.1 | -11.1 | 98.1 | 1.2 | 0.7 | 0.5 |
| Croissants | 66.9 | 10.6 | 22.5 | -11.9 | 92.2 | 1.9 | 5.9 | -4.0 |
| Ready-to-heat vegetables | 69.6 | 8.3 | 22.1 | -13.8 | 97.5 | 1.7 | 0.80 | 0.9 |
| Soft drinks | 67.4 | 9.1 | 23.5 | -14.4 | 98.4 | 0.5 | 1.1 | -0.6 |
| Instant sauces | 71.6 | 6.2 | 22.3 | -16.1 | 99.2 | 0.2 | 0.6 | -0.4 |
| Plant-based meat substitutes | 77.5 | 2.4 | 20.2 | -17.8 | 99.7 | 0.1 | 0.2 | -0.1 |
| Plant-based cheese substitutes (e.g. tofu) | 77.7 | 1.6 | 20.7 | -19.1 | 99.7 | 0.1 | 0.2 | -0.1 |

All p values for difference between cohorts <0.0001 (adjusted for age and sex).

**Supplementary Table 3.** Association of demographic and socioeconomic factors with self-rated changes in ultra-processed food (UPF) consumption during the Italian lockdown following the COVID-19 pandemic (March 9 – May 3, 2020) in the ALT RISCOVID-19 and Moli-LOCK study cohorts by means of adjusted regression coefficients (β) with 95% confidence interval (95%CI), Italy 2020

|  | **ALT RISCOVID-19 (n=1,491)** | | | | | **Moli-LOCK Study (n=1,501)** | | | |
| --- | --- | --- | --- | --- | --- | --- | --- | --- | --- |
|  | % | UPF score (mean, SD) | β (95%CI)^1^ | β (95%CI)^2^ | % | | UPF score (mean, SD) | β (95%CI)^1^ | β (95%CI)^2^ |
| Age groups (y) |  |  |  |  |  | |  |  |  |
| 18-39 | 29.7 | -0.36 (5.21) | Ref. | Ref. | - | | - | - | - |
| 40-55 | 38.2 | -0.77 (5.83) | -0.39 (-1.07, 0.28) | -0.68 (-1.45, 0.09) | - | | - | - | - |
| 56-65 | 20.2 | -1.21 (5.34) | -0.87 (-1.67, -0.08) | -1.26 (-2.18, -0.34) | - | | - | - | - |
| 66-75 | 9.9 | -1.86 (5.25) | -1.73 (-2.75, -0.71) | -2.21 (-3.37, -1.05) | - | | - | - | - |
| >75 | 2.0 | -2.00 (4.66) | -2.18 (-4.24, -0.11) | -2.76 (-4.89, -0.64) | - | | - | - | - |
| Age groups (y) |  |  |  |  |  | |  |  |  |
| 49-55 | - | - | - | - | 9.2 | | 0.46 (2.24) | Ref. | Ref. |
| 56-65 | - | - | - | - | 28.6 | | 0.49 (1.69) | 0.01 (-0.29, 0.31) | -0.06 (-0.36, 0.25) |
| 66-70 | - | - | - | - | 22.9 | | 0.29 (1.58) | -0.16 (-0.47, 0.14) | -0.22 (-0.54, 0.11) |
| >70 | - | - | - | - | 39.4 | | 0.15 (1.18) | -0.29 (-0.58, -0.01) | -0.34 (-0.69, -0.02) |
| Sex |  |  |  |  |  | |  |  |  |
| Women | 63.2 | -1.29 (5.83) | Ref. | Ref. | 56.1 | | 0.42 (1.68) | Ref. | Ref. |
| Men | 36.8 | -0.15 (4.78) | 1.29 (0.71, 1.88) | 1.15 (0.56, 1.75) | 43.9 | | 0.17 (1.36) | -0.22 (-0.38, -0.06) | -0.25 (-0.43,-0.07) |
| Geographical areas* |  |  |  |  |  | |  |  |  |
| Northern | 34.7 | -0.09 (4.88) | Ref. | Ref. | 0.00 | | - | - | - |
| Central | 13.3 | -0.56 (5.49) | -0.33 (-1.22, 0.56) | -0.33 (-1.23, 0.57) | 0.1 | | - | - | - |
| Southern and Islands | 48.7 | -1.53 (5.89) | -1.48 (-2.09, -0.87) | -1.26 (-1.93, -0.60) | 99.9 | | - | - | - |
| Living area |  |  |  |  |  | |  |  |  |
| >200,000 inhabitants | 22.7 | -0.46 (4.96) | Ref. | Ref. | - | | - | - | - |
| <200,000 inhabitants | 23.7 | -0.52 (4.90) | -0.12 (-0.93, 0.69) | 0.28 (-0.56, 1.12) | - | | - | - | - |
| <50,000 inhabitants | 24.1 | -0.86 (5.42) | -0.51 (-1.31, 0.30) | 0.07 (-0.80, 0.93) | - | | - | - | - |
| Villages/rural areas | 29.4 | -1.47 (6.30) | -1.15 (-1.92, -0.37) | -0.36 (-1.21, 0.50) | - | | - | - | - |
| Living area | - | - | - | - |  | |  |  |  |
| Urban | - | - | - | - | 84.5 | | 0.34 (1.60) | Ref. | Ref. |
| Villages/rural areas | - | - | - | - | 15.5 | | 0.16 (0.10) | -0.20 (-0.42, 0.01) | -0.22 (-0.44, 0.004) |
| Educational level |  |  |  |  |  | |  |  |  |
| Up to lower secondary | 4.0 | -2.17 (6.48) | Ref. | Ref. | 32.8 | | 0.21 (1.33) | Ref. | Ref. |
| Upper secondary | 26.1 | -1.04 (5.84) | 1.06 (-0.43, 2.56) | 0.66 (-0.86, 2.19) | 46.4 | | 0.41 (1.52) | 0.18 (-0.002, 0.36) | 0.14 (-0.08, 0.35) |
| Postgraduate | 69.9 | -0.73 (5.29) | 1.39 (-0.04, 2.83) | 0.57 (-0.99, 2.13) | 20.8 | | 0.25 (1.91) | 0.01 (-0.22, 0.23) | -0.13 (-0.44, 0.18) |
| Household income (EUR/year) |  |  |  |  |  | |  |  |  |
| ≤10,000 | 4.8 | -1.35 (6.01) | Ref. | Ref. | 5.3 | | 0.43 (1.02) | Ref. | Ref. |
| >10,000≤25,000 | 22.3 | -1.52 (6.30) | 0.05 (-1.35, 1.45) | -0.32 (-1.73, 1.09) | 32.5 | | 0.25 (1.63) | -0.17 (-0.54, 0.20) | -0.27 (-0.65, 0.11) |
| >25,000≤40,000 | 22.0 | -0.62 (5.61) | 1.10 (-0.31, 2.50) | 0.50 (-0.93, 1.93) | 36.6 | | 0.19 (1.61) | -0.22 (-0.59, 0.14) | -0.44 (-0.84, -0.03) |
| >40,000≤60,000 | 14.1 | -0.48 (4.96) | 1.28 (-0.21, 2.77) | 0.44 (-1.09, 1.98) | 11.2 | | 0.39 (1.63) | -0.01 (-0.42, 0.41) | -0.22 (-0.68, 0.25) |
| >60,000 | 19.7 | -0.29 (4.57) | 1.51 (0.05, 2.96) | 0.53 (-1.01, 2.06) | 2.9 | | 0.68 (1.16) | 0.24 (-0.33, 0.81) | 0.08 (-0.53, 0.69) |
| Unknown | 17.1 | -1.20 (5.39) | 0.48 (-0.95, 1.92) | -0.03 (-1.49, 1.42) | 11.5 | | 0.62 (1.33) | 0.14 (-0.27, 0.55) | -0.004 (-0.44, 0.43) |
| Marital status |  |  |  |  |  | |  |  |  |
| Married/in couple | 63.4 | -0.77 (5.35) | Ref. | Ref. | 82.8 | | 0.34 (1.51) | Ref. | Ref. |
| Unmarried | 27.3 | -0.87 (5.63) | -0.54 (-1.25, 0.18) | -0.06 (-0.85, 0.73) | 3.9 | | -0.17 (2.42) | -0.59 (-1.00, -0.18) | -0.62 (-1.08, -0.16) |
| Divorced | 7.2 | -1.14 (6.13) | -0.11 (-1.20, 0.99) | 0.10 (-1.04, 1.24) | 3.5 | | 0.40 (1.86) | 0.01 (-0.42, 0.44) | -0.05 (-0.50, 0.40) |
| Widower | 2.1 | -2.84 (5.45) | -1.24 (-3.22, 0.75) | -0.79 (-2.80, 1.21) | 9.8 | | 0.23 (1.31) | -0.07 (-0.35, 0.21) | -0.09 (-0.45, 0.28) |
| Number of cohabitants |  |  |  |  |  | |  |  |  |
| None | 10.8 | -1.70 (6.23) | Ref. | Ref. | 10.4 | | 0.14 (1.44) | Ref. | Ref. |
| 1 | 28.7 | -0.73 (5.06) | 1.00 (0.01, 1.99) | 0.95 (-0.14, 2.03) | 48.3 | | 0.23 (1.45) | 0.12 (-0.15, 0.39) | -0.03 (-0.39, 0.35) |
| 2 | 24.7 | -0.48 (5.25) | 1.01 (-0.01, 2.02) | 1.14 (0.02, 2.26) | 22.4 | | 0.53 (1.56) | 0.37 (0.07, 0.67) | 0.21 (-0.19, 0.62) |
| >2 | 35.8 | -0.99 (5.72) | 0.45 (-0.52, 1.42) | 0.58 (-0.54, 1.70) | 18.9 | | 0.33 (1.81) | 0.10 (-0.22, 0.43) | -0.10 (-0.53, 0.33) |
| Occupational class |  |  |  |  |  | |  |  |  |
| Professional/managerial | 59.8 | -0.68 (5.17) | Ref. | Ref. | 27.3 | | 0.34 (1.54) | Ref. | Ref. |
| Skilled non-manual | 22.3 | -0.74 (5.71) | -0.01 (-0.68, 0.70) | 0.08 (-0.68, 0.83) | 39.4 | | 0.37 (1.64) | 0.02 (-0.18, 0.21) | -0.05 (-0.27, 0.17) |
| Skilled manual | 3.1 | -1.22 (4.53) | -0.92 (-2.54, 0.70) | -0.29 (-2.04, 1.45) | 6.8 | | 0.19 (1.34) | -0.07 (-0.41, 0.27) | -0.10 (-0.49, 0.29) |
| Partly skilled/unskilled | 1.8 | -0.74 (7.52) | -0.53 (-2.62, 1.56) | 0.07 (-2.06, 2.19) | 10.5 | | 0.00 (1.55) | -0.32 (-0.60, -0.03) | -0.37 (-0.70, -0.04) |
| Unemployed/unclassified | 13.0 | -1.90 (6.30) | -1.35 (-2.21, -0.49) | -0.92 (-1.84, 0.01) | 16.0 | | 0.37 (1.40) | -0.09 (-0.35, 0.16) | -0.20 (-0.51, 0.11) |

*Numbers do not add up to 100% due to missing data.

^1^Multivariable-adjusted linear regression including age and sex.

^2^ Multivariable-adjusted linear regression including age, sex, geographical area (not in the Moli-LOCK cohort), living area, educational level, household income, marital status, number of cohabitants, occupational class.

**Supplementary Table 4.** Association of lockdown-induced factors with self-reported changes in ultra-processed food (UPF) consumption during the Italian lockdown following the COVID-19 pandemic (March 9 – May 3, 2020) in the ALT RISCOVID-19 and Moli-LOCK study cohorts by means of adjusted regression coefficients (β) with 95% confidence interval (95%CI), Italy 2020

|  | **ALT RISCOVID-19 (n=1,491)** | | | **Moli-LOCK Study (n=1,501)** | | |
| --- | --- | --- | --- | --- | --- | --- |
|  | % | UPF score (mean, SD) | β (95%CI) ^1^ | % | UPF score (mean, SD) | β (95%CI) ^2^ |
| Work type during lockdown* |  |  |  |  |  |  |
| Usual working | 24.7 | -0.61 (5.12) | Ref. | 6.3 | 0.22 (1.63) | Ref. |
| Home working | 34.4 | -0.64 (5.39) | 0.04 (-0.69, 0.78) | 12.5 | 0.53 (2.22) | 0.26 (-0.13, 0.64) |
| Work interruption | 12.6 | -1.63 (6.36) | -0.68 (-1.67, 0.32) | 5.9 | 0.40 (1.68) | 0.18 (-0.27, 0.62) |
| Work reduction | 8.2 | -0.49 (5.92) | 0.08 (-1.04, 1.19) | 1.1 | 1.37 (1.63) | 1.31 (0.49, 2.13) |
| Job loss | 1.7 | 0.12 (5.93) | 1.02 (-1.22, 3.26) | 0.3 | -0.25 (1.89) | -0.15 (-1.70, 1.40) |
| Retired/housewife | 13.8 | -1.77 (5.16) | -0.12 (-1.29, 1.04) | 71.2 | 0.25 (1.39) | 0.21 (-0.16, 0.57) |
| Income support |  |  |  |  |  |  |
| No | 65.7 | -0.89 (0.17) | Ref. | 85.9 | 0.28 (1.55) | Ref. |
| Yes | 34.3 | -0.82 (0.24) | -0.54 (-1.14, 0.06) | 14.1 | 0.49 (1.54) | 0.08 (-0.16, 0.32) |
| Income reduction* |  |  |  |  |  |  |
| No | 51.4 | -0.58 (5.00) | Ref. | 83.9 | 0.29 (1.55) | Ref. |
| Yes | 38.5 | -1.02 (5.98) | 0.02 (-0.59, 0.62) | 13.1 | 0.47 (1.54) | 0.06 (-0.19, 0.32) |
| Sport activity during lockdown* |  |  |  |  |  |  |
| Unchanged | 28.5 | -0.45 (4.89) | Ref. | 34.5 | 0.18 (1.12) | Ref. |
| Increased | 16.8 | -1.38 (5.11) | -0.98 (-1.82, -0.13) | 1.7 | -0.48 (1.94) | -0.59 (-1.21, 0.03) |
| Decreased | 53.5 | -0.92 (5.86) | -0.41 (-1.05, 0.23) | 45.7 | 0.36 (1.66) | 0.14 (-0.04, 0.32) |
| Physical activity during lockdown * |  |  |  |  |  |  |
| Unchanged | 85.6 | -0.92 (5.41) | Ref. | 94.6 | 0.29 (1.56) | Ref. |
| Increased | 7.8 | -0.16 (5.70) | 0.91 (-0.12, 1.94) | 2.8 | 0.74 (1.25) | 0.28 (-0.20, 0.76) |
| Decreased | 6.6 | -0.99 (6.25) | 0.08 (-1.04, 1.20) | 2.6 | 0.51 (1.55) | 0.23 (-0.26, 0.72) |
| Diagnosis of chronic diseases during lockdown* |  |  |  |  |  |  |
| No | 91.8 | -0.89 (5.47) | Ref. | 98.5 | 0.32 (1.55) | Ref. |
| Yes | 7.2 | -0.25 (5.63) | 1.02 (-0.04, 2.09) | 1.3 | -0.30 (1.42) | -0.70 (-1.38, -0.02) |
| Drug use during lockdown* |  |  |  |  |  |  |
| No | 93.5 | -0.86 (5.44) | Ref. | 98.7 | 0.31 (1.56) | Ref. |
| Yes | 5.6 | -1.08 (6.51) | -0.01 (-1.21, 1.19) | 1.3 | 0.31 (0.75) | 0.05 (-0.64, 0.75) |

*Numbers do not add up to 100% due to missing data.

^1^Multivariable-adjusted linear regression including age, sex, geographical area, number of cohabitants, occupational class.

^2^Multivariable-adjusted linear regression including age, sex, living area, household income, marital status, occupational class.

**Supplementary Table 5.** Association of diet-related behaviours with self-rated changes in ultra-processed food (UPF) consumption during the Italian lockdown following the COVID-19 pandemic (March 9 – May 3, 2020) in the ALT RISCOVID-19 and Moli-LOCK study cohorts by means of adjusted regression coefficients (β) with 95% confidence interval (95%CI), Italy 2020

|  | **ALT RISCOVID-19 (n=1,491)** | | | **Moli-LOCK Study (n=1,501)** | | |
| --- | --- | --- | --- | --- | --- | --- |
|  | % | UPF score (mean, SD) | β (95%CI) ^1^ | % | UPF score (mean, SD) | β (95%CI)^2^ |
| Body weight |  |  |  |  |  |  |
| Unchanged | 47.5 | -1.46 (4.93) | Ref. | 52.6 | 0.12 (1.38) | Ref. |
| Increased | 39.3 | 0.53 (5.56) | 1.93 (1.34, 2.51) | 36.0 | 0.78 (1.58) | 0.60 (0.43, 0.77) |
| Decreased | 11.9 | -2.98 (6.13) | -1.61 (-2.49, -0.74) | 10.9 | -0.27 (1.84) | -0.41 (-0.67, -0.16) |
| Take away food |  |  |  |  |  |  |
| Unchanged | 58.6 | -0.79 (5.12) | Ref. | 93.1 | 0.28 (1.54) | Ref. |
| Increased | 19.4 | 0.40 (5.79) | 0.63 (-0.09, 1.36) | 3.9 | 0.81 (1.48) | 0.45 (0.05, 0.86) |
| Reduced | 21.0 | -2.03 (5.71) | -1.43 (-2.13, -0.72) | 2.3 | 0.77 (1.88) | 0.37 (-0.15, 0.89) |
| Time spent on home  food preparation |  |  |  |  |  |  |
| Unchanged | 42.4 | -1.01 (5.15) | Ref. | 55.8 | -0.11 (1.48) | Ref. |
| Increased | 54.5 | -0.70 (5.67) | 0.29 (-0.29, 0.86) | 42.8 | 0.88 (1.45) | 0.96 (0.80, 1.11) |
| Reduced | 2.0 | -1.67 (6.73) | -0.64 (-2.63, 1.34) | 1.2 | -0.89 (1.78) | -0.80 (-1.49,-0.11) |
| Number of daily meals |  |  |  |  |  |  |
| Unchanged | 74.0 | -1.45 (5.10) | Ref. | 85.4 | 0.18 (1.42) | Ref. |
| Increased | 22.9 | 1.22 (6.08) | 2.83 (2.18, 3.49) | 12.3 | 1.39 (1.77) | 1.12 (0.89, 1.35) |
| Reduced | 2.9 | -2.39 (6.15) | -1.02 (-2.64, 0.60) | 1.9 | - 1.00 (2.37) | -1.25 (-1.81,-0.69) |
| Food supplements use |  |  |  |  |  |  |
| Unchanged | 83.7 | -0.80 (5.32) | Ref. | 97.4 | 0.29 (1.54) | Ref. |
| Increased | 11.9 | -1.57 (5.74) | -0.35 (-1.20, 0.50) | 1.6 | 1.17 (1.83) | 0.73 (0.10, 1.36) |
| Reduced | 2.1 | -1.35 (8.29) | -0.53 (-2.46, 1.40) | 0.4 | 0.83 (1.72) | 0.13 (-1.11, 1.37) |
| Water intake |  |  |  |  |  |  |
| Unchanged | 68.2 | -0.86 (4.97) | Ref. | 90.9 | 0.28 (1.52) | Ref. |
| Increased | 27.2 | -0.83 (6.51) | 0.003 (-0.63, 0.64) | 7.3 | 0.45 (1.84) | 0.11 (-0.19, 0.42) |
| Reduced | 4.2 | -1.21 (6.36) | -0.46 (-1.86, 0.94) | 1.3 | 1.15 (1.50) | 0.66 (-0.03, 1.34) |
| Food budget |  |  |  |  |  |  |
| Unchanged | 45.7 | -1.27 (4.96) | Ref. | 65.6 | 0.16 (1.51) | Ref. |
| Increased | 43.0 | -0.19 (5.72) | 0.82 (0.22, 1.43) | 17.9 | 0.70 (1.63) | 0.48 (0.27, 0.69) |
| Reduced | 10.3 | -1.84 (6.43) | -0.33 (-1.28, 0.62) | 15.5 | 0.43 (1.53) | 0.24 (0.02, 0.46) |
| Short food supply chain |  |  |  |  |  |  |
| Unchanged | 44.1 | -0.84 (5.00) | Ref. | 68.0 | 0.19 (1.56) | Ref. |
| Increased | 44.1 | -0.92 (5.77) | 0.08 (-0.52, 0.67) | 22.3 | 0.61 (1.46) | 0.41 (0.22, 0.60) |
| Reduced | 10.9 | -0.57 (6.12) | 0.16 (-0.77, 1.09) | 8.3 | 0.52 (1.48) | 0.32 (0.03, 0.61) |
| Long food supply chain |  |  |  |  |  |  |
| Unchanged | 42.7 | -0.92 (4.69) | Ref. | 66.7 | 0.19 (1.50) | Ref. |
| Increased | 27.4 | -0.18 (6.15) | 0.59 (-0.08, 1.27) | 8.1 | 0.57 (1.69) | 0.40 (0.11, 0.69) |
| Reduced | 29.2 | -1.42 (5.82) | -0.18 (-0.85, 0.49) | 23.7 | 0.59 (1.54) | 0.40 (0.21, 0.59) |
| Organic food |  |  |  |  |  |  |
| Unchanged | 85.2 | -0.64 (5.22) | Ref. | 97.5 | 0.29 (1.55) | Ref. |
| Increased | 9.3 | - 2.01 (7.08) | -1.35 (-2.30, -0.41) | 1.1 | 0.81 (1.22) | 0.45 (-0.32, 1.21) |
| Reduced | 2.8 | -2.71 (6.05) | -2.16 (-3.84, -0.48) | 0.4 | 1.67 (0.82) | 1.29 (0.06, 2.54) |
| Local food |  |  |  |  |  |  |
| Unchanged | 71.2 | -0.57 (5.21) | Ref. | 86.5 | 0.22 (1.48) | Ref. |
| Increased | 22.1 | -1.69 (6.04) | -0.98 (-1.65, -0.31) | 7.4 | 0.70 (1.22) | 0.51 (0.21, 0.81) |
| Reduced | 4.8 | -1.12 (6.23) | -0.36 (-1.65, 0.93) | 5.2 | 1.04 (2.52) | 0.68 (0.33, 1.04) |
| Pre-prepared meals |  |  |  |  |  |  |
| Unchanged | 64.5 | 0.03 (4.45) | Ref. | 95.5 | 0.28 (1.53) | Ref. |
| Increased | 7.4 | 2.35 (5.50) | 2.05 (1.06, 3.04) | 2.9 | 1.37 (1.91) | 0.98 (0.51, 1.45) |
| Reduced | 22.5 | - 4.36 (6.44) | -4.34 (-4.97, -3.71) | 0.7 | 0.09 (1.37) | -0.36 (-1.27, 0.55) |
| Long shelf life food |  |  |  |  |  |  |
| Unchanged | 67.1 | - 0.49 (4.61) | Ref. | 91.0 | 0.26 (1.41) | Ref. |
| Increased | 20.3 | 0.96 (5.80) | 1.41 (0.76, 2.06) | 7.3 | 0.98 (2.54) | 0.59 (0.29, 0.90) |
| Reduced | 11.5 | -6.09 (6.30) | -5.54 (-6.37, -4.72) | 0.9 | -0.07 (2.61) | -0.64 (-1.45, 0.17) |

*Numbers do not add up to 100% due to missing data.

^1^Multivariable-adjusted linear regression including age, sex, geographical area, number of cohabitants, occupational class.

^2^Multivariable-adjusted linear regression including age, sex, living area, household income, marital status, occupational class.

**Moli-LOCK Study Investigators**

**Principal Investigator:** Licia Iacoviello^1,2^

**Research team:** Licia Iacoviello (chairperson)^1,2^, Marialaura Bonaccio^1^, Americo Bonanni^1^, Francesca Bracone^1^, Chiara Cerletti^1^, Simona Costanzo^1^, Giovanni de Gaetano^1^, Maria Benedetta Donati^1^, Simona Esposito^1^, Alessandro Gialluisi^1^, Mariarosaria Persichillo^1^, Emilia Ruggiero^1^.

**Data management:** Simona Costanzo^1^, Marco Olivieri^4^.

**Data Analysis:** Marialaura Bonaccio^1^, Simona Costanzo^1^, Augusto Di Castelnuovo^3^, Alessandro Gialluisi^1^, Emilia Ruggiero^1^.

**Interviewers (telephone):** Mariarosaria Persichillo (coordinator) ^1^, Francesca Bracone^1^, Francesca De Lucia^4^, Alessandro Del Giudice, Cristiana Mignogna^2^, Teresa Panzera^1^, Jonathan Squillante.

1 Department of Epidemiology and Prevention, IRCCS Neuromed, Pozzilli, Italy

2 Department of Medicine and Surgery, University of Insubria, Varese-Como, Italy

3 Mediterranea, Cardiocentro, Napoli, Italy

4 Associazione Cuore-Sano ONLUS, Campobasso, Italy
